# Sequence analysis of the combinations of work shifts and absences in health care – comparison of two years of administrative data

**DOI:** 10.1186/s12912-022-01160-1

**Published:** 2022-12-30

**Authors:** Oxana Krutova, Laura Peutere, Jenni Ervasti, Mikko Härmä, Marianna Virtanen, Annina Ropponen

**Affiliations:** 1 Finnish Institute of Occupational Health, P.O.Box 18, Helsinki, 00032 Finland; 2 School of Educational Sciences and Psychology, University of Eastern Finland, Joensuu, Finland; 3 CNS, Division of Insurance Medicine, Karolinska Institutet, Stockholm, Sweden

**Keywords:** Shift work, Absence, Sequence analysis, Longitudinal, Health care, Employees

## Abstract

**Background:**

In health care, the shift work is arranged as irregular work shifts to provide operational hours for 24/7 care. We aimed to investigate working hour trends and turnover in health care via identification of time-related sequences of work shifts and absences among health care employees. The transitions between the work shifts (i.e., morning, day, evening, and night shifts), and absences (days off and other leaves) over time were analyzed and the predictors of change in irregular shift work were quantified.

**Methods:**

A longitudinal cohort study was conducted using employer-owned payroll-based register data of objective and day-to-day working hours and absences of one hospital district in Finland from 2014 to 2019 (*n* = 4931 employees). The working hour data included start and end of work shifts, any kind of absence from work (days off, sickness absence, parental leave), and employee’s age, and sex. Daily work shifts and absences in 2014 and 2019 were used in sequence analysis. Generalized linear model was used to estimate how each identified sequence cluster was associated with sex and age.

**Results:**

We identified four sequence clusters: “Morning” (60% in 2014 and 56% in 2019), “Varying shift types” (22% both in 2014 and 2019), “Employee turnover” (13% in 2014 and 3% in 2019), and “Unstable employment (5% in 2014 and 19% in 2019). The analysis of transitions from one cluster to another between 2014 and 2019 indicated that most employees stayed in the same clusters, and most often in the “Varying shift types” (60%) and “Morning” (72%) clusters. The majority of those who moved, moved to the cluster “Morning” in 2019 from “Employee turnover” (43%), “Unstable employment” (46%) or “Varying shift types” (21%). Women were more often than men in the clusters “Employee turnover” and “Unstable employment”, whereas older employees were more often in “Morning” and less often in the other cluster groups.

**Conclusion:**

Four clusters with different combinations of work shifts and absences were identified. The transition rates between work shifts and absences with five years in between indicated that most employees stayed in the same clusters. The likelihood of a working hour pattern characterized by “Morning” seems to increase with age.

**Supplementary Information:**

The online version contains supplementary material available at 10.1186/s12912-022-01160-1.

## Introduction

Shift work, organized as irregular work shifts including morning, day, evening, and night shifts is common in health care due to the provision of 24/7 care [1, 2]. Furthermore, irregular working hours in health care may include rotating shifts across consecutive days, even without sufficient rest between shifts, and during scheduled time off [3]. Shift work, even without but especially with night work, may influence health and well-being [4, 5], e.g., due to sleep disorders [6–8], occupational stress [9] and chronic fatigue [10]. Shift work is also known to be linked with many chronic conditions such as gastrointestinal symptoms [11] or with circadian strain, and the risk of cardiovascular disease and cancer mortality, especially among women working rotating night shifts for at least 5 years [12–14]. Studies that have identified trends and turnover of irregular working hours in health care are rare [15], i.e. it is not known whether employees remain in stressful irregular working hours or whether they transfer to day work due to aging or other reasons. Selection out of shift work may be linked e.g. to healthy worker effect [16].

An emergent challenge is to address the complexity of irregular working hours in health care when investigating the combinations of work shifts and different types of absences [1, 15, 17]. Irregular working hour patterns are often a consequence of the need to fulfil the changing and irregular needs of service production while accounting for legal restrictions. Staffing in health care is planned with shift scheduling which requires optimal assignment of the required working hours according to available personal resources, legal and safety requirements, and the requests of the employees [18].

The earlier register-based working hour studies using payroll data have investigated the working hour characteristics in shift work to obtain unbiased understanding of irregular working hours with various health outcomes [19–23]. However, these previous studies have mostly investigated only a few working hour characteristics at a time [21–23] or various working hour characteristics separately [17, 20, 24] and studies of combinations of the irregular working hours are rare [1, 15]. Hence, a need exists to conduct studies with objective data of irregular working hours to identify combinations of work shifts and absences, their mutual associations, and changes over time. Ageing might increase transition from shift work to day work [24, 25] but also other kinds of transitions, such as work disability or turnover (leaving the employer or the entire health care sector) [26–28]. New employees are needed to substitute the absences and turnover [26, 27].

To investigate working hour trends and turnover in health care, we aimed to identify time-related sequences of work shifts and absences in irregular working hours among health care employees. Further on, we investigate the transitions between work shifts and absences (i.e., between morning, day, evening, night shifts, days off, or leaves) over time and quantify the predictors for change. We hypothesized that the incidence of day work would increase, and shift work decrease based on turnover, health worker effect and ageing [26–28].

## Methods

This study utilized employer-owned register data of working hours and absences of one hospital district in 2014 and 2019. The full sample included 7997 employees i.e., having an employee identification code in 2014 and 8231 employees in 2019. The final analytical sample was restricted to 4931 health care workers who had a shift work contract in their first working day in 2014 and had remained in the register also in 2019 (i.e., had at least one work shift or leave during each year).

The data were obtained from the shift scheduling program Titania® (CGI Finland). The payroll-based working hour data included information on the work unit and work time contract (shift work and possible part-time contracts) start and end of all daily work shifts and absences including days off, sickness absences, vacations and other leaves, [17]. The work shifts were classified into early morning shift (starting before 06:00, but not categorized as a night shift), morning shift (starting after 03:00 and ending before 18:00); day shift (starting after 08:00 and ending before 18:00); evening shift (≥ 3 h of work between 18:00 and 02:00 and not categorized as a night shift); and night shift (≥ 3 h of work between 23:00 and 06:00) as has been done before [1, 17]. The work shifts can be slightly overlapping, i.e., a shift may fit in to more than one of the shift categories. Therefore, the work shifts were prioritized for their health-related exposure in which each shift was assigned as either night (highest priority), evening or day (lowest priority).

The reasons for absence were available from the working hour data and included annual vacation, day off, sick leave, parental leaves (i.e., maternity/paternity leave or temporary care leave (e.g., when a child is sick)), and other leaves (i.e., employment interruptions, leaves due to training, unpaid leaves, treatment, or examination approved by the employer for working time or other date of suspension, and sudden unpaid absence (e.g., due to strike)). The observations which had missing work shift or absence code due to different specific work stops (e.g., normal non-working hours) or absences (e.g., leaves due to training) were collapsed into one category named “missing” (2014 – *n* = 20,754 days, 2019 – *n* = 44,060 days, 0.89% of all days in the register for 2014 year and 1.9% of all days in the register for 2019 year).

The data was organized in the accuracy of calendar days: each employee would potentially have 365 observations per year and has been classified to one (first mentioned) status per calendar day. The years 2014 and 2019 were selected to control the number of observations in the analyses. The maximum available data in each year was 365 days for 2014, and 358 days for 2019 between 1 January and 31 December and having at least one work shift or absence. The data comprised of employer owned employment information why no ethical approval was required for the study.

### Statistical analysis

Statistical analyses were conducted with R (version 4.0.5) and the TraMineR package for an analysis of sequences of work shifts (early morning shift, morning shift, day shift, evening shift or night shift) and absences (e.g., vacation, day off, sick leave, parental leave, or other leave). The date was used as the order variable (delta = 1 day). We utilized cluster analysis with optimal matching spell (Optimal Matching, OM) to identify different clusters of employees who had similar sequences of work shifts and absences. Separate analyses were conducted for years 2014 and 2019. The optimal number of clusters was selected based on an agglomerative hierarchical clustering using the obtained distance matrix. We used agnes() to make a hierarchical clustering with the Ward method. The cluster solution was retained after examining the dendrogram of the clustering tree for years 2014 and 2019.

We estimated the transition rates for sequence objects to obtain information about the most frequent state changes observed in the data together with an assessment of the stability of each state [29]. Furthermore, the Generalized Linear Model (GLM) were used to analyze the associations between cluster group, sex, and age. As GLM (binomial logistic regression) provides log odds ratios, we calculates odds ratios (OR) based on exp(log odds ratios) function and 95% confidence intervals (CI) based on exp(log odds ratios ± 1.96 × SE) function.

Then we investigated further one of the clusters named “Varying shift types” that was assumed to represent the irregular working hours in health care. For this cluster group, we carried out a separate sequence analysis.

## Results

### Cluster groups

**Fig. 1:**
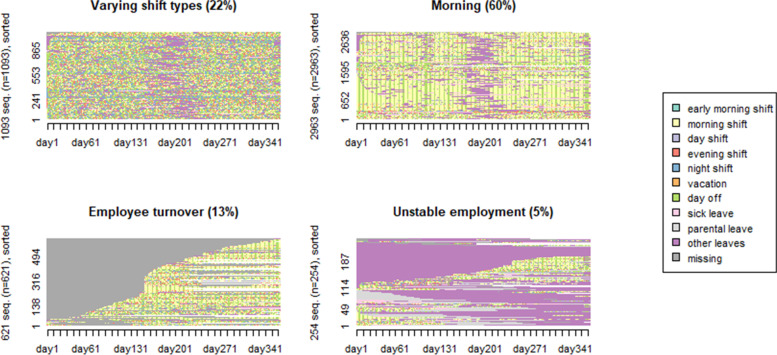
The full sequence index plots for day-to-day work shifts and absences in 2014 (sorted by beginning 1st of January) for the identified four cluster groups. The number of employees at the y-axis and days from 1st of January 2014 to 31st of December 2014 at the x-axis

**Fig. 2:**
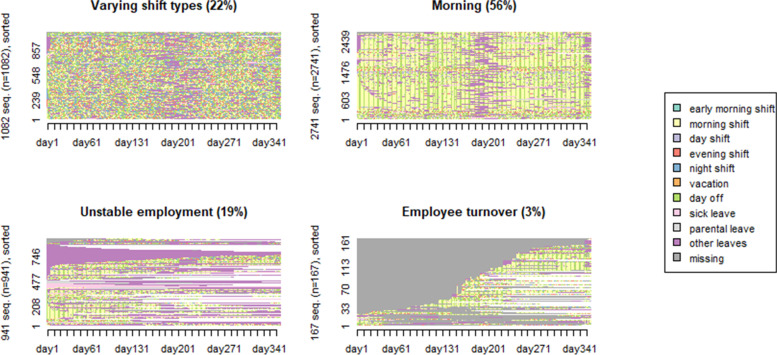
The full sequence index plots for day-to-day work shifts and absences in 2019 (sorted by beginning 1st of January) for the identified four cluster groups. The number of employees at the y-axis and days from 1st of January 2019 to 31st of December 2019 at the x-axis

The final sample of 4931 employees in 2014 had a mean age 41.6 (SD 10.6) years and of them, 85% were women. The clusters with combinations of work shift and absence for years 2014 and 2019 are shown in Figs. 1 and 2.

Each employee represents one sequence. The sequence analysis showed that both in 2014 and in 2019 four cluster groups of employees (sequences) could be identified (Figs. 1 and 2). The cluster groups were named as:

1. “Morning” (60% in 2014 and 56% in 2019), that included dominantly morning shifts, but only few absence episodes. The employees had on average 151–155 morning shifts and 90–94 days off during the year (Supplemental Table 1A), and a relatively stable employment.
2. “Varying shift types” (22% both in 2014 and 2019) was distinctive due to a combination of various work shifts as, for example, morning shift (69 shifts), day shifts (8–10), evening shifts (38–44), night shifts (46–47) and days off (100–110 days), and a relatively stable employment, i.e., few absence episodes.
3. “Employee turnover” (13% in 2014 and 3% in 2019) included a large proportion of new employees who entered the employment later in the year and/or left the job during the same year.
4. “Unstable employment” (5% in 2014 and 19% in 2019) implied that employees had dominantly morning shift when at work, but considerable absence from work. This cluster of employees was distinctive due to a larger number of days in sick leave, parental leave, other absences parallel to rare morning shifts (28–42 shifts) and having rather small number of days off (23–35 days).

The analysis of transitions from one cluster group in 2014 to another in 2019 indicated that most employees stayed in the same clusters, and most often in the “Varying shift types” (60%) and “Morning” cluster groups (72%) (Table 1). However, among those who moved from one cluster in 2014 to another one in 2019, the majority moved to the cluster “Morning” from such clusters as “Employee turnover” (43%), “Unstable employment” (46%) or “Varying shift types” (21%). Also transitions from “Employee turnover” and from “Unstable employment” to “Varying shift types” were rather frequent (25% and 19%, respectively).

**Table 1:** Comparison of transitions from cluster groups in 2014 to cluster groups 2019

Cluster groups in 2014	Cluster groups in 2019							
	Varying types		Morning		Employee turnover		Unstable employment	
	N	%	N	%	N	%	N	%
Varying shift types	657	60	227	21	24	2	185	17
Morning	221	7	2131	72	78	3	533	18
Employee turnover	157	25	265	43	57	9	142	23
Unstable employment	47	19	118	46	8	3	81	32

The transition rates of the work shifts and absences within 2014 and 2019 indicated a relatively high probability of staying in the same statuses for night shift, sick leave, parental leave and other leaves (e.g., the transition analysis shows ‘transitions’ from night shift to night shift, from sick leave to sick leave etc.) (Supplemental Table 1B). Instead, a higher likelihood of transitions was detected from evening shift to morning shift or from day shift to morning shift. In 2014 and 2019, the transitions from evening shift to morning shift were about the same (0.49 and 0.41, respectively) as well as transitions from day shift to morning shift (0.43 and 0.40).

Table 2 contains the regression associations between cluster group, sex, and age. Women were more likely to belong to cluster groups “Employee turnover” and “Unstable employment” (Table 2). Older age groups had increased likelihood of belonging to the “Morning” group. Younger age groups were more likely to belong to the “Varying shift types”, “Employee turnover” and “Unstable employment” clusters. Full descriptive statistics for the clusters is given in Supplemental Table 1D.

**Table 2:** Odds ratios and 95% CI for the associations age and sex with identified clusters “Varying shift types “, “Morning “, “Employee turnover” and “Unstable employment”

	Varying shift types		Morning		Employee turnover		Unstable employment	
	OR	95% CI	OR	95% CI	OR	95% CI	OR	95% CI
**2014**								
Sex/woman (reference Man)	1.16	0.96–1.42	0.85	0.72–1.01	**0.69**	**0.55–0.87**	**3.91**	**2.31–7.24**
Age (reference < 30 years)								
31–45 years	**0.70**	**0.59–0.84**	**3.26**	**2.77–3.85**	**0.22**	**0.18–0.27**	**1.37**	**1.01–1.90**
46–55 years	**0.58**	**0.48–0.71**	**7.05**	**5.87–8.48**	**0.08**	**0.06–0.10**	**0.18**	**0.10–0.31**
> 56 years	**0.44**	**0.34–0.58**	**8.18**	**6.43–10.45**	**0.05**	**0.03–0.08**	**0.59**	**0.34–0.97**
**2019**								
Sex/woman (reference Man)	0.95	0.79–1.15	1.07	0.91–1.25	**0.72**	**0.49–1.09**	1.02	0.84–1.25
Age (reference < 30 years)								
31–45 years	**0.83**	**0.63–1.09**	**2.03**	**1.56–2.66**	**0.39**	**0.25–0.62**	**0.68**	**0.52–0.91**
46–55 years	**0.58**	**0.44–0.78**	**4.64**	**3.53–6.14**	**0.07**	**0.03–0.14**	**0.32**	**0.24–0.44**
> 56 years	**0.33**	**0.25–0.45**	**4.13**	**3.14–5.46**	**0.29**	**0.17–0.48**	**0.60**	**0.45–0.80**

Statistically significant OR with 95%CI in boldface

### The cluster “Varying shift types”

**Fig. 3:**
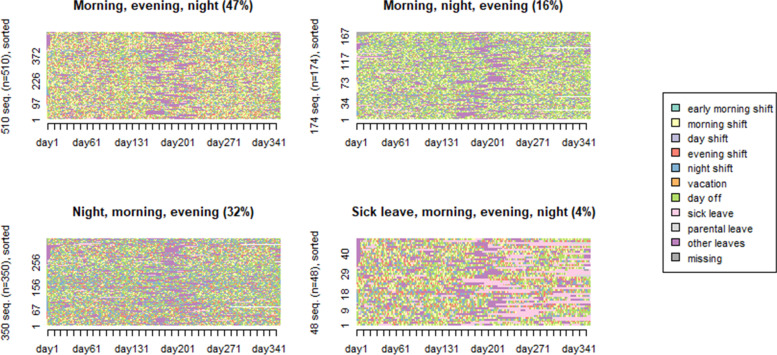
Full-sequence index plots as classified for four types of transition sequences as sorted by beginning (1st of January 2019) for the cluster “Varying shift types”. The number of employees at the y-axis and days from 1st of January 2019 to 31st of December 2019 at the x-axis

We conducted a more detailed analysis on the group “Varying shift types” because it was assumed to represent the irregularity of working hours and absences in health care. Figure 3 shows the more detailed sequences for the cluster group “Varying shift types” in 2019. Within this cluster group, the four clusters of sequences were named according to the most frequent work shifts presented in each cluster, i.e., ‘Morning, evening, night’ represents a combination of morning, evening, and night shifts. The other clusters were ‘Morning, night, evening’, ‘Night, morning, evening’ and ‘Sick leave, morning, evening, night’.

The morning shift remained the most frequent work shift for clusters Morning, evening, night and Morning, night, evening. For the same clusters, a combination of evening and night shifts remained also rather frequent with the difference in prevalence either evening or night shifts. The cluster Sick leave, morning, evening, night was like Morning, evening, night, however, sick leaves were the most frequent for this cluster. For the cluster Night, morning, evening, the most frequent work shift was night shift in combination with evening and morning shifts. Analysis of mean time spent in each work shift or leave is given in Supplemental Table 1C.

Table 3 contains the transition rates for sequence objects, which allows considering the most frequent state changes together with an assessment of the stability of each state. Transition rate 1 would mean that the transition always exists between two states, whereas the closer the zero the transition rates go, the less likely it is that a transition would take place. We estimated transition rates for the cluster group “Varying shift types” (estimations are not given here). The transitions from morning shift most probably occurred to morning shift (0.40), and less likely to evening shift (0.15) or night shift (0.09). Transitions from evening shift were most likely to morning shift (0.37), less to evening shift (0.27) or night shift (0.12). The transitions from night shift to night shift were very likely (0.97). Furthermore, we estimated transitions between work shifts for each of the clusters (Table 3). If the morning shift was the main shift, transitions probably occurred from morning shift to morning shift or from evening shift to morning shift for cluster 1 Morning, evening, night and cluster 2 Morning, night, evening.

**Table 3:** Estimated transition rates in 2019 between work shifts and absences for four types of transition sequences for the cluster “Varying shift types” (*n* = 1082)

From/to	Early morning shift	Morning shift	Day shift	Evening shift	Night shift	Vacation	Day off	Sick leave	Parental leave	Other leaves
**Cluster 1 Morning, evening, night**										
Early morning shift	0.00	0.67	0.00	0.33	0.00	0.00	0.00	0.00	0.00	0.00
Morning shift	0.00	0.43	0.05	0.16	0.06	0.00	0.29	0.01	0.00	0.02
Day shift	0.00	0.49	0.16	0.10	0.05	0.00	0.18	0.01	0.00	0.01
Evening shift	0.00	0.43	0.05	0.25	0.09	0.00	0.16	0.01	0.00	0.01
Night shift	0.00	0.01	0.01	0.01	0.97	0.00	0.00	0.00	0.00	0.00
Vacation	0.00	0.49	0.17	0.02	0.00	0.00	0.24	0.01	0.00	0.07
Day off	0.00	0.21	0.05	0.17	0.06	0.00	0.46	0.01	0.00	0.04
Sick leave	0.00	0.09	0.02	0.04	0.03	0.00	0.14	0.66	0.00	0.03
Parental leave	0.00	0.00	0.00	0.00	0.00	0.00	0.05	0.00	0.92	0.03
Other leaves	0.00	0.03	0.01	0.02	0.01	0.00	0.05	0.00	0.00	0.88
**Cluster 2 Morning, night, evening**										
Early morning shift	0.00	0.00	0.00	0.00	0.00	0.00	1.00	0.00	0.00	0.00
Morning shift	0.00	0.44	0.02	0.07	0.16	0.00	0.28	0.01	0.00	0.02
Day shift	0.00	0.40	0.12	0.04	0.09	0.00	0.32	0.01	0.00	0.03
Evening shift	0.00	0.35	0.02	0.20	0.15	0.00	0.26	0.01	0.00	0.02
Night shift	0.00	0.01	0.01	0.01	0.97	0.00	0.00	0.00	0.00	0.00
Vacation	0.00	0.00	0.00	0.00	0.00	0.00	0.00	0.00	0.00	0.00
Day off	0.00	0.22	0.02	0.05	0.06	0.00	0.60	0.01	0.00	0.04
Sick leave	0.00	0.04	0.01	0.01	0.03	0.00	0.12	0.76	0.00	0.03
Parental leave	0.00	0.00	0.00	0.00	0.08	0.00	0.08	0.00	0.85	0.00
Other leaves	0.00	0.04	0.01	0.01	0.01	0.00	0.08	0.00	0.00	0.86
**Cluster 3 Night, morning, evening**										
Early morning shift	0.00	0.00	0.00	0.00	0.00	0.00	0.00	0.00	0.00	0.00
Morning shift	0.00	0.34	0.02	0.19	0.15	0.00	0.28	0.01	0.00	0.02
Day shift	0.00	0.33	0.09	0.12	0.13	0.00	0.30	0.02	0.00	0.01
Evening shift	0.00	0.29	0.02	0.31	0.19	0.00	0.17	0.01	0.00	0.01
Night shift	0.00	0.01	0.01	0.01	0.98	0.00	0.00	0.00	0.00	0.00
Vacation	0.00	1.00	0.00	0.00	0.00	0.00	0.00	0.00	0.00	0.00
Day off	0.00	0.14	0.02	0.15	0.12	0.00	0.53	0.01	0.00	0.04
Sick leave	0.00	0.03	0.00	0.03	0.05	0.00	0.12	0.72	0.00	0.04
Parental leave	0.00	0.00	0.00	0.00	0.00	0.00	0.14	0.00	0.77	0.09
Other leaves	0.00	0.02	0.00	0.02	0.02	0.00	0.07	0.01	0.00	0.87
**Cluster 4 Sick leave, morning, evening, night**										
Early morning shift	0.00	0.00	0.00	0.00	0.00	0.00	0.00	0.00	0.00	0.00
Morning shift	0.00	0.37	0.02	0.20	0.07	0.00	0.30	0.02	0.00	0.02
Day shift	0.00	0.46	0.10	0.11	0.05	0.00	0.24	0.02	0.00	0.02
Evening shift	0.00	0.33	0.02	0.34	0.09	0.00	0.18	0.02	0.00	0.01
Night shift	0.00	0.01	0.00	0.01	0.97	0.00	0.00	0.00	0.00	0.00
Vacation	0.00	0.00	0.00	0.00	0.00	0.00	0.00	0.00	0.00	0.00
Day off	0.00	0.17	0.02	0.18	0.05	0.00	0.52	0.03	0.00	0.04
Sick leave	0.00	0.02	0.00	0.01	0.01	0.00	0.04	0.92	0.00	0.01
Parental leave	0.00	0.00	0.00	0.00	0.00	0.00	0.00	0.00	1.00	0.00
Other leaves	0.00	0.02	0.00	0.02	0.01	0.00	0.06	0.02	0.00	0.87

For the cluster 3 Night, morning, evening, transitions from evening shift or from day shift to night shift were higher than for other clusters. The last cluster (Sick leave, morning, evening, night) was distinctive due to more frequent sick leaves in combination with frequent morning, evening, and night shifts. Transitions from evening shifts to evening shifts were more frequent ones for the last cluster as well.

## Discussion

This study with objective register data of working hours of one hospital district with 4931 employees in 2014 and 2019 aimed to identify working hour trends and turnover in health care. Another aim was to investigate the transitions over time between the work shift types and absences and quantify the predictors of changes. We identified four clusters: “Morning” (60% in 2014 and 56% in 2019), “Varying shift types” (22% both in 2014 and 2019), “Employee turnover” (13% in 2014 and 3% in 2019) and “Unstable employment” (5% in 2014 and 19% in 2019). The largest cluster group, “Morning”, i.e., the combination of morning shifts and day offs was most common, whereas the combination between various work shifts and days offs (cluster group “Varying shift types”) was also rather frequent being in line with earlier indications that around 20–25% of employees in health care work shifts including night work [3, 30–32]. The transition rates between work shifts and absences from 2014 to 2019 indicated that most employees stayed in the same cluster groups, and most often in the “Varying shift types” and “Morning” cluster groups. However, among those who moved from one cluster in 2014 to another one in 2019, the majority moved to clusters “Morning” and “Varying shift types”. In addition, transitions from “Varying shift types” and “Morning” to “Employee turnover” or “Unstable employment” were frequent.

The findings yielded mixed support for the hypothesis. The observed transitions rates did not support the assumption that among those health care employees who stayed in the same workplace, the work shift and absence patterns would have been changed considerably over the five years, instead they were stable. However, while assessing the role of age for cluster groups, older age was associated with a lower likelihood of belonging to the “Varying shift types” cluster group and a greater likelihood of belonging to the “Morning” cluster group indicating that older employees and thus those who had longer time span spent as shift workers tend to switch from shift work to daywork.

Our results are in line with earlier findings of a larger dataset linked with the current study but from earlier years and not applying a sequence analysis [17], in which the correlations between working hour characteristics (e.g. length of the working hours, time of the day, shift intensity, and social aspects of working hours) within individuals were relatively stable between 2008 and 2013. Furthermore, we also identified that staying in the same status had a relatively high probability for night shift, sick leave, parental leave and other leaves, being in line with an earlier study [1].

With regard to absence from work, studies with survey data suggest that intention to leave the employment in health care might be due to high effort–reward imbalance, reward frustration, poor salary and promotion prospects and lack of esteem [33], work-related fatigue, work conditions, and health [34], but also that the demands of nursing work, the inconvenience of shift work/working hours and uncertain work status [35] and restricted professional autonomy play a role [36]. For sickness absences, the surveyed factors that may explain absences and being out of employment have been fatigue, poor inter-shift recovery, perceived workload, obstructive sleep apnea and marital status [37], shiftwork (mostly nightwork), ageing and work-related stress [38] and two-shift and three-shift rotations, as well as fixed night shifts [39]. In this study, we had register data only, hence restricting the investigation of influential factors to age and sex. We found that women were less likely to belong to the cluster “Employee turnover” and more likely to belong to the cluster “Unstable employment” which somewhat supports the earlier findings of precarious work in health care [4, 28, 40, 41].

Women in health care may have somewhat precarious work [42], i.e., they have interruptions in their working careers due to various reasons that could be further investigated in other studies. However, a cautious interpretation could be that both at workplace level but also at societal level, support for career planning within health care or for prevention o interruptions in working should be provided. Instead, older employees had a greater likelihood of belonging to the “Morning” cluster but a lower probability to belong to the other cluster groups which also seems to follow previous survey results [38].

For the separately investigated “Varying shift types” cluster group in 2019, a combination of morning, evening, and night shifts was common. For example, morning shift remained the basic work shift. At the same time, a combination of evening and night shifts remained also rather frequent with the difference in the prevalence of evening or night shifts. Finally, more frequent sick leaves in combination with frequent morning, evening, and night shifts were rather frequent for the cluster “Varying shift types” in 2019. Our results are in the line with a previous study from the same dataset [15] showing relatively regular morning- and evening-oriented shift work with weekends off and highly irregular working hours with night and weekend shifts under short contracts.

As a strength of this study, we utilized the objective, pay-roll based employer owned working hour data without recall bias, covering an entire hospital district in Finland. The data also allowed comparison of two years. Another strength related to the use of sequence analysis is that it takes the advantage of the rich, day-to-day data to make a full description of work shifts and absences within two years to identify patterns. Furthermore, sequence analysis in combination with cluster analysis allowed synthesizing enormous volume of information, consisting of various sequences of work shifts and absences, into relatively homogeneous groups. On the other hand, applied sequence analysis method has certain limitations, i.e., one-dimensionality of categories and elements, which aggregate a sequence. However, until now, this kind of analyses with objective working hour data have been rare [17] and focused on comparison of shift starting and ending times [1, 2] or estimating an outcome [15]. A limitation might be the lack of influential factors (predictors) assessed in this study. One might assume that organization of working hours [2, 15, 43], other organizational issues (such as managers, culture) [44, 45] or even health or workability of employees [46] might play a role in the clusters of work shifts and absences. This should be addressed in further studies although the sequences of this study indicate the combinations of work shifts and absences but less why they cluster. Irregular working hours with various clusters of work shifts and absences are common in general in the service sector besides health care [1, 47, 48]. Furthermore, we lacked the knowledge of diagnoses for sickness absence, which restricts us from comparisons of sickness absences due to underlying illness. Based on earlier studies of irregular working hour in the health care in the Nordic countries [1, 2, 19], we may assume these results to be generalizable to them, but less perhaps in other countries with different welfare systems for absences.

### Conclusion

In a large administrative data of hospital employees, four cluster groups with different combinations of work shifts and absences were identified. After five years, two of the cluster groups were rather stable, characterized by being employed in health care with varying work shift combinations. Two cluster groups were indicative for unstable employment due to high variation of various absences or not being employed. The transition rates between work shifts and absences between five years indicated that most employees stayed in the same cluster groups, but older employees tended to move to day work. Both administrative data and data-driven approach for identification of working hours patterns and absences would be potential both for further studies and for follow-up at hospitals to understand turnover of employees.

## Supplementary Information

**Additional file 1: Supplemental Table 1A.** Mean number of days/work shifts for cluster groups in 2014 and 2019. **Supplemental Table 1B.** Transition rates used for the sequence object (2014 and 2019). **Supplemental Table 1C.** Frequency of days/work shifts for the cluster “Varying shift types” in 2019. **Supplemental Table 1D.** Frequencies and percentages for sex and mean with standard deviation (sd) for age for cluster groups in 2014 and 2019.

## Data Availability

The data that support the findings of this study are available from the hospital district of Southwest Finland but restrictions apply to the availability of these data, which were used under license for the current study, and so are not publicly available. Data are however available from the authors upon reasonable request and with permission of the hospital district of Southwest Finland.

## Abbreviations

CI: Confidence intervals
GLM: Generalized Linear Model
OM: Optimal matching
OR: Odds ratio
SE: Standard Error

## References

[1] Garde AH, Harris A, Vedaa Ø, Bjorvatn B, Hansen J, Hansen ÅM, Kolstad HA, Koskinen A, Pallesen S, Ropponen A (2019). Working hour characteristics and schedules among nurses in three nordic countries – a comparative study using payroll data. BMC Nurs.

[2] Garde AH, Begtrup L, Bjorvatn B, Bonde JP, Hansen J, Hansen ÅM, Härmä M, Jensen MA, Kecklund G, Kolstad HA (2020). How to schedule night shift work in order to reduce health and safety risks. Scand J Work Environ Health.

[3] Trinkoff A, Geiger-Brown J, Brady B, Lipscomb J, Muntaner C (2006). How long and how much are nurses now working?. Am J Nurs.

[4] Steinmetz S, de Vries DH, Tijdens KG (2014). Should I stay or should I go? The impact of working time and wages on retention in the health workforce. Hum Resour Health.

[5] Bernstrøm VH, Alves DE, Ellingsen D, Ingelsrud MH (2019). Healthy working time arrangements for healthcare personnel and patients: a systematic literature review. BMC Health Serv Res.

[6] Zverev YP, Misiri HE (2009). Perceived effects of rotating shift work on nurses’ sleep quality and duration. Malawi Med J.

[7] Chan MF (2009). Factors associated with perceived sleep quality of nurses working on rotating shifts. J Clin Nurs.

[8] Ferri P, Guadi M, Marcheselli L, Balduzzi S, Magnani D, Di Lorenzo R (2016). The impact of shift work on the psychological and physical health of nurses in a general hospital: a comparison between rotating night shifts and day shifts. Risk Manag Healthc Policy.

[9] Lin P-C, Chen C-H, Pan S-M, Chen Y-M, Pan C-H, Hung H-C, Wu M-T (2015). The association between rotating shift work and increased occupational stress in nurses. J Occup Health.

[10] Muecke S (2005). Effects of rotating night shifts: literature review. J Adv Nurs.

[11] Nojkov B, Rubenstein JH, Chey WD, Hoogerwerf WA (2010). The impact of rotating Shift work on the prevalence of irritable bowel syndrome in nurses. Official J Am Coll Gastroenterol | ACG.

[12] Schernhammer ES, Laden F, Speizer FE, Willett WC, Hunter DJ, Kawachi I, Colditz GA (2001). Rotating night shifts and risk of breast Cancer in women participating in the Nurses’ Health Study. JNCI: J Natl Cancer Inst.

[13] Gu F, Han J, Laden F, Pan A, Caporaso NE, Stampfer MJ, Kawachi I, Rexrode KM, Willett WC, Hankinson SE (2015). Total and cause-specific mortality of U.S. nurses working rotating night shifts. Am J Prev Med.

[14] Wegrzyn LR, Tamimi RM, Rosner BA, Brown SB, Stevens RG, Eliassen AH, Laden F, Willett WC, Hankinson SE, Schernhammer ES (2017). Rotating night-shift work and the risk of breast Cancer in the Nurses’ Health Studies. Am J Epidemiol.

[15] Rosenström T, Härmä M, Kivimäki M, Ervasti J, Virtanen M, Hakola T, Koskinen A, Ropponen A (2021). Patterns of working hour characteristics and risk of sickness absence among shift-working hospital employees: a data-mining cohort study. Scand J Work Environ Health.

[16] Baillargeon J (2001). Characteristics of the healthy worker effect. Occup Med.

[17] Härmä M, Ropponen A, Hakola T, Koskinen A, Vanttola P, Puttonen S, Sallinen M, Salo P, Oksanen T, Pentti J (2015). Developing register-based measures for assessment of working time patterns for epidemiologic studies. Scand J Work Environ Health.

[18] Ropponen A, Vanttola P, Koskinen A, Hakola T, Puttonen S, Härmä M (2017). Effects of modifications to the health and social sector’s collective agreement on the objective characteristics of working hours. Ind Health.

[19] Larsen AD, Ropponen A, Hansen J, Hansen ÅM, Kolstad HA, Koskinen A, Härmä MI, Garde AH (2020). Working time characteristics and long-term sickness absence among danish and finnish nurses: a register-based study. Int J Nurs Stud.

[20] Ropponen A, Koskinen A, Puttonen S, Härmä M (2019). Exposure to working-hour characteristics and short sickness absence in hospital workers: a case-crossover study using objective data. Int J Nurs Stud.

[21] Vedaa Ø, Harris A, Erevik EK, Waage S, Bjorvatn B, Sivertsen B, Moen BE, Pallesen S (2019). Short rest between shifts (quick returns) and night work is associated with work-related accidents. Int Arch Occup Environ Health.

[22] Vedaa Ø, Pallesen S, Erevik EK, Svensen E, Waage S, Bjorvatn B, Sivertsen B, Harris A (2019). Long working hours are inversely related to sick leave in the following 3 months: a 4-year registry study. Int Arch Occup Environ Health.

[23] Dall’Ora C, Ball J, Redfern O, Recio-Saucedo A, Maruotti A, Meredith P, Griffiths P (2019). Are long nursing shifts on hospital wards associated with sickness absence? A longitudinal retrospective observational study. J Nurs Manag.

[24] Karhula K, Hakola T, Koskinen A, Lallukka T, Ojajärvi A, Puttonen S, Oksanen T, Rahkonen O, Ropponen A, Härmä M. Ageing shift workers’ sleep and working-hour characteristics after implementing ergonomic shift-scheduling rules. J Sleep Res. 2021;30(4):e13227. 10.1111/jsr.13227 PMC8365717 33166038

[25] Strandell R (2020). Care workers under pressure - A comparison of the work situation in swedish home care 2005 and 2015. Health Soc Care Community.

[26] Chen J, Davis LS, Davis KG, Pan W, Daraiseh NM (2011). Physiological and behavioural response patterns at work among hospital nurses. J Nurs Manag.

[27] Schnelle JF, Schroyer LD, Saraf AA, Simmons SF (2016). Determining nurse aide staffing requirements to provide Care based on Resident workload: a Discrete Event Simulation Model. J Am Med Dir Assoc.

[28] Xu G, Zeng X, Wu X. Global prevalence of turnover intention among intensive care nurses: a meta-analysis. Nurs Crit Care. 2021;1-8. 10.1111/nicc.12679 34261191

[29] Gabadinho A, Ritschard G, Müller NS, Studer M (2011). Analyzing and visualizing state sequences in R with TraMineR. J Stat Softw.

[30] Han K, Trinkoff AM, Geiger-Brown J (2014). Factors associated with work-related fatigue and recovery in hospital nurses working 12-hour shifts. Workplace Health Saf.

[31] Härmä M, Karhula K, Puttonen S, Ropponen A, Koskinen A, Ojajärvi A, Kivimäki M (2019). Shift work with and without night work as a risk factor for fatigue and changes in sleep length: a cohort study with linkage to records on daily working hours. J Sleep Res.

[32] Härmä M, Kecklund G (2010). Shift work and health — how to proceed?. Scand J Work Environ Health.

[33] Li J, Galatsch M, Siegrist J, Müller BH, Hasselhorn HM (2011). Reward frustration at work and intention to leave the nursing profession—prospective results from the european longitudinal NEXT study. Int J Nurs Stud.

[34] Liu Y, Wu LM, Chou PL, Chen MH, Yang LC, Hsu HT (2016). The influence of work-related fatigue, work conditions, and personal characteristics on intent to leave among New Nurses. J Nurs Scholarsh.

[35] Flinkman M, Laine M, Leino-Kilpi H, Hasselhorn HM, Salanterä S (2008). Explaining young registered finnish nurses’ intention to leave the profession: a questionnaire survey. Int J Nurs Stud.

[36] Fochsen G, Sjögren K, Josephson M, Lagerström M (2005). Factors contributing to the decision to leave nursing care: a study among swedish nursing personnel. J Nurs Adm Manag.

[37] Sagherian K, Unick GJ, Zhu S, Derickson D, Hinds PS, Geiger-Brown J (2017). Acute fatigue predicts sickness absence in the workplace: a 1-year retrospective cohort study in paediatric nurses. J Adv Nurs.

[38] Conway PM, Campanini P, Sartori S, Dotti R, Costa G (2008). Main and interactive effects of shiftwork, age and work stress on health in an italian sample of healthcare workers. Appl Ergon.

[39] Bernstrøm VH, Houkes I (2020). Shift work and sickness absence at a norwegian hospital: a longitudinal multilevel study. Occup Environ Med.

[40] Prado-Gascó V, Giménez-Espert MDC, De Witte H (2021). Job insecurity in nursing: a bibliometric analysis. Int J Environ Res Public Health.

[41] Moloney W, Boxall P, Parsons M, Cheung G (2018). Factors predicting registered Nurses’ intentions to leave their organization and profession: a job demands-resources framework. J Adv Nurs.

[42] Hult M, Halminen O, Mattila-Holappa P, Kangasniemi M (2022). Health and work well-being associated with employment precariousness among permanent and temporary nurses: a cross-sectional survey. Nordic J Nurs Res.

[43] Sallinen M, Kecklund G (2010). Shift work, sleep, and sleepiness - differences between shift schedules and systems. Scand J Work Environ Health.

[44] Lundstrom T, Pugliese G, Bartley J, Cox J, Guither C (2002). Organizational and environmental factors that affect worker health and safety and patient outcomes. Am J Infect Control.

[45] Aloisio LD, Coughlin M, Squires JE (2021). Individual and organizational factors of nurses’ job satisfaction in long-term care: a systematic review. Int J Nurs Stud.

[46] Kowalczuk K, Krajewska-Kułak E, Sobolewski M. Working excessively and burnout among nurses in the context of sick leaves. Front Psychol. 2020;11:285. 10.3389/fpsyg.2020.00285 PMC7052176 32158416

[47] Nätti J, Oinas T, Härmä M, Anttila T, Kandolin I (2014). Combined effects of shiftwork and individual working time control on long-term sickness absence: a prospective study of finnish employees. J Occup Environ Med.

[48] Ropponen A, Hakola T, Hirvonen M, Koskinen A, HÄRmÄ M: Working hour characteristics in the Finnish retail sector – a registry study on objective working hour data. Ind Health. 2022. advpub. 10.2486/indhealth.2021-0138 PMC9171130 34690253

