# Supplementary material for: Sequence analysis of the combinations of work shifts and absences in health care – comparison of two years of administrative data
Source: BMC Nurs. 2022 Dec 30;21:376. doi: 10.1186/s12912-022-01160-1 (PMC9801614; doi:10.1186/s12912-022-01160-1)
Supplement: Supplementary file 1 — Additional file 1: Supplemental Table 1A. Mean number of days/work shifts for cluster groups in 2014 and 2019. Supplemental Table 1B. Transition rates used for the sequence object (2014 and 2019). Supplemental Table 1C. Frequency of days/work shifts for the cluster “Varying shift types” in 2019. Supplemental Table 1D. Frequencies and percentages for sex and mean with standard deviation (sd) for age for cluster groups in 2014 and 2019. [file 12912_2022_1160_MOESM1_ESM.docx]

**Supplemental material**

**Supplemental Table 1A.** Mean number of days/work shifts for cluster groups in 2014 and 2019

|  | **2014** | | | | | **2019** | | | | |
| --- | --- | --- | --- | --- | --- | --- | --- | --- | --- | --- |
|  | **All clusters** | **Varying shift types** | **Morning** | **Employee turnover** | **Unstable employment** | **All clusters** | **Varying shift types** | **Morning** | **Employee turnover** | **Unstable employment** |
|  | **Mean** | **Mean** | **Mean** | **Mean** | **Mean** | **Mean** | **Mean** | **Mean** | **Mean** | **Mean** |
| early morning shifts | 0 | 0 | 0 | 0 | 0 | 0 | 0 | 0 | 0 | 0 |
| morning shifts | 115 | 69 | 151 | 56 | 28 | 110 | 69 | 155 | 34 | 42 |
| day shifts | 12 | 8 | 16 | 5 | 5 | 12 | 10 | 15 | 4 | 5 |
| evening shifts | 17 | 44 | 10 | 11 | 4 | 16 | 38 | 11 | 5 | 6 |
| night shifts | 13 | 47 | 3 | 6 | 3 | 11 | 46 | 1 | 2 | 3 |
| vacation days | 4 | 3 | 6 | 1 | 1 | 2 | 0 | 3 | 0 | 1 |
| days off | 85 | 110 | 90 | 41 | 23 | 83 | 100 | 94 | 27 | 35 |
| sick leave days | 12 | 10 | 15 | 3 | 8 | 16 | 11 | 13 | 2 | 29 |
| parental leave days | 4 | 0 | 2 | 1 | 42 | 2 | 0 | 1 | 1 | 10 |
| other leaves (days) | 57 | 46 | 56 | 8 | 229 | 66 | 53 | 55 | 16 | 121 |

**Supplemental Table 1B.** Transition rates used for the sequence object (2014 and 2019).

|  | **2014** | | | | | | | | |
| --- | --- | --- | --- | --- | --- | --- | --- | --- | --- |
|  | morning shift | day shift | evening shift | night shift | vacation | day off | sick leave | parental leave | other leaves |
| early morning shift | 0.14 | 0.01 | 0.01 | 0.01 | 0.00 | 0.29 | 0.02 | 0.00 | 0.01 |
| morning shift | 0.63 | 0.03 | 0.04 | 0.02 | 0.01 | 0.24 | 0.01 | 0.00 | 0.02 |
| day shift | 0.43 | 0.32 | 0.03 | 0.01 | 0.01 | 0.18 | 0.01 | 0.00 | 0.02 |
| evening shift | 0.50 | 0.03 | 0.24 | 0.08 | 0.00 | 0.13 | 0.01 | 0.00 | 0.01 |
| night shift | 0.01 | 0.01 | 0.00 | 0.99 | 0.00 | 0.00 | 0.00 | 0.00 | 0.00 |
| vacation | 0.41 | 0.11 | 0.03 | 0.01 | 0.03 | 0.33 | 0.02 | 0.00 | 0.06 |
| day off | 0.27 | 0.04 | 0.08 | 0.03 | 0.03 | 0.50 | 0.01 | 0.00 | 0.04 |
| sick leave | 0.07 | 0.01 | 0.01 | 0.01 | 0.00 | 0.08 | 0.81 | 0.00 | 0.02 |
| parental leave | 0.00 | 0.00 | 0.00 | 0.00 | 0.00 | 0.00 | 0.00 | 0.99 | 0.01 |
| other leaves | 0.05 | 0.01 | 0.01 | 0.00 | 0.00 | 0.04 | 0.00 | 0.00 | 0.89 |
|  | **2019** | | | | | | | | |
| early morning shift | 0.13 | 0.02 | 0.00 | 0.00 | 0.00 | 0.28 | 0.01 | 0.00 | 0.03 |
| morning shift | 0.64 | 0.03 | 0.04 | 0.02 | 0.00 | 0.24 | 0.01 | 0.00 | 0.02 |
| day shift | 0.41 | 0.32 | 0.03 | 0.02 | 0.00 | 0.20 | 0.01 | 0.00 | 0.02 |
| evening shift | 0.41 | 0.04 | 0.27 | 0.08 | 0.00 | 0.18 | 0.01 | 0.00 | 0.01 |
| night shift | 0.01 | 0.01 | 0.01 | 0.97 | 0.00 | 0.00 | 0.00 | 0.00 | 0.00 |
| vacation | 0.57 | 0.06 | 0.02 | 0.00 | 0.03 | 0.21 | 0.02 | 0.00 | 0.09 |
| day off | 0.29 | 0.04 | 0.07 | 0.02 | 0.02 | 0.51 | 0.01 | 0.00 | 0.05 |
| sick leave | 0.05 | 0.01 | 0.01 | 0.01 | 0.00 | 0.06 | 0.85 | 0.00 | 0.01 |
| parental leave | 0.00 | 0.00 | 0.00 | 0.00 | 0.00 | 0.00 | 0.00 | 0.99 | 0.01 |
| other leaves | 0.05 | 0.01 | 0.01 | 0.00 | 0.00 | 0.05 | 0.00 | 0.00 | 0.88 |

Supplemental Table 1C. Frequency of days/work shifts for the cluster “Varying shift types” in 2019

|  | **All** | **Morning, evening, night** | **Morning, night, evening** | **Night, morning, evening** | **Sick leave, morning, evening, night** |
| --- | --- | --- | --- | --- | --- |
| **Number of** | **Mean** | **Mean** | **Mean** | **Mean** | **Mean** |
| early morning shifts | 0 | 0 | 0 | 0 | 0 |
| morning shifts | 69 | 87 | 71 | 44 | 55 |
| day shifts | 10 | 14 | 7 | 4 | 4 |
| evening shifts | 38 | 45 | 16 | 38 | 45 |
| night shifts | 46 | 34 | 41 | 67 | 29 |
| vacation days | 0 | 0 | 0 | 0 | 0 |
| days off | 100 | 98 | 130 | 100 | 89 |
| sick leave days | 11 | 8 | 11 | 10 | 65 |
| parental leave days | 0 | 0 | 0 | 0 | 1 |
| other leave days | 53 | 51 | 56 | 53 | 54 |

**Supplemental Table 1D.** Frequencies and percentages for sex and mean with standard deviation (sd) for age for cluster groups in 2014 and 2019

|  | **2014** | | | | | | | |
| --- | --- | --- | --- | --- | --- | --- | --- | --- |
|  | Varying shift types | | Morning | | Employee turnover | | Unstable employment | |
|  | **n** | **%** | **n** | **%** | **n** | **%** | **n** | **%** |
| Man | 150 | 14 | 449 | 15 | 125 | 20 | 13 | 5 |
| Woman | 943 | 86 | 2,514 | 85 | 496 | 80 | 241 | 95 |
|  | **mean** | **sd** | **mean** | **sd** | **mean** | **sd** | **mean** | **sd** |
| Age (years) | 39.72 | 10.33 | 44.71 | 9.62 | 32.29 | 9.61 | 36.80 | 8.98 |
|  | **2019** | | | | | | | |
|  | **n** | **%** | **n** | **%** | **n** | **%** | **n** | **%** |
| Man | 173 | 16 | 391 | 14 | 33 | 20 | 140 | 15 |
| Woman | 909 | 84 | 2,350 | 86 | 134 | 80 | 801 | 85 |
|  | **mean** | **sd** | **mean** | **sd** | **mean** | **sd** | **mean** | **sd** |
| Age (years) | 43.69 | 9.94 | 48.58 | 9.79 | 42.34 | 13.74 | 45.09 | 11.97 |
